# An integrated risk and vulnerability assessment framework for climate change and malaria transmission in East Africa

**DOI:** 10.1186/s12936-016-1600-3

**Published:** 2016-11-11

**Authors:** Esther Achieng Onyango, Oz Sahin, Alex Awiti, Cordia Chu, Brendan Mackey

**Affiliations:** 1 Centre for Environment and Population Health, Griffith University, School of Environment, 170 Kessels Road, Nathan, 4111 Australia; 2 School of Engineering, Griffith University, Gold Coast, 4222 Australia; 3 East African Institute, Aga Khan University East Africa, 2nd Parklands Avenue, Nairobi, 00100 Kenya; 4 Griffith Climate Change Response Program, Griffith University, Gold Coast, 4222 Australia

**Keywords:** Integrated risk and vulnerability assessment, Climate change impact on malaria transmission, Systems approach, Climate change and malaria risk, East Africa

## Abstract

**Background:**

Malaria is one of the key research concerns in climate change-health relationships. Numerous risk assessments and modelling studies provide evidence that the transmission range of malaria will expand with rising temperatures, adversely impacting on vulnerable communities in the East African highlands. While there exist multiple lines of evidence for the influence of climate change on malaria transmission, there is insufficient understanding of the complex and interdependent factors that determine the risk and vulnerability of human populations at the community level. Moreover, existing studies have had limited focus on the nature of the impacts on vulnerable communities or how well they are prepared to cope. In order to address these gaps, a systems approach was used to present an integrated risk and vulnerability assessment framework for studies of community level risk and vulnerability to malaria due to climate change.

**Results:**

Drawing upon published literature on existing frameworks, a systems approach was applied to characterize the factors influencing the interactions between climate change and malaria transmission. This involved structural analysis to determine influential, relay, dependent and autonomous variables in order to construct a detailed causal loop conceptual model that illustrates the relationships among key variables. An integrated assessment framework that considers indicators of both biophysical and social vulnerability was proposed based on the conceptual model.

**Conclusions:**

A major conclusion was that this integrated assessment framework can be implemented using Bayesian Belief Networks, and applied at a community level using both quantitative and qualitative methods with stakeholder engagement. The approach enables a robust assessment of community level risk and vulnerability to malaria, along with contextually relevant and targeted adaptation strategies for dealing with malaria transmission that incorporate both scientific and community perspectives.

## Background

It is estimated that at least 3.3 billion people globally are at risk of malaria infection. The disease is responsible for over half a million deaths each year, mostly (90%) in sub-Saharan Africa. Current climate change projections estimate an increase in the population at risk of malaria by 1.6 million by 2030 and 1.8 million by 2050 [1, 2]. This risk is significant in East Africa whereby rising temperatures and changes in other climate conditions are projected to expand the transmission range of malaria into geographic areas where communities were previously unexposed to the disease [3]. Understanding the extent to which local communities are vulnerable to this risk and how well they cope, is necessary to inform policies and interventions for risk management.

Vulnerability is determined in part by changes in land use and associated socio-economic and cultural factors at the community level, which exacerbate climate change impacts on malaria transmission. Previous vulnerability assessments have largely overlooked the influence of these socio-economic and cultural factors, instead emphasizing the biophysical influences on malaria transmission. While the evidence is abundant on increased risk of malaria as a result of changing climate, more robust understanding is needed of environmental, cultural and socioeconomic factors that influence malaria transmission at the community and household levels. This requires an integrated approach, which considers climate along with the contribution of socio-economic and cultural factors in order to explore current and future risks and vulnerabilities to malaria transmission.

While there are general guidelines on conducting integrated risk and vulnerability assessments, there is not one accepted method or approach in use that reflects specific contexts and the availability of data. This paper will provide a review of literature in climate change and malaria transmission in East Africa, and use this previous research to identify key variables in malaria transmission in order to construct a systems conceptual model and an integrated risk and vulnerability assessment framework.

### The threat of malaria in a warmer world: climate change and malaria research in East Africa

Warming over the African continent is faster than the global average [4]. Projections for the next century show that most areas of the continent will exceed the 2 °C threshold by the last two decades of this century under medium scenarios and that under high scenarios this will happen by mid-century and reach between 3 and 6 °C by the end of the century [3]. The malaria mosquito and parasite are both sensitive to changes in climate and climate variability and the projected rising temperatures and changes in rainfall patterns will create favourable conditions for mosquito breeding in many areas [3, 4]. In East Africa, climate scenarios suggest longer malaria transmission seasons and geographic expansion of the disease into highland areas [5–8]. According to published literature, the earliest malaria-climate connection in the East African highlands was identified in the 1980s when there was a series of malaria epidemics connected to increases and anomalies in mean monthly maximum temperatures and increase in rainfall in the highlands [9–12]. Since then, the frequency and size of epidemics increased with serious outbreaks in 1995, 1998 and 2002, corresponding to climate variations such as a significant increase (≥3 °C) in mean temperatures [9], high rainfall [10], drought and El Nino events [9, 13–17]. Concurrently, increasing human population and intensified agricultural activities in the highlands has led to land use changes that in turn have enhanced vector production. At local scales, these changes in land cover, along with differences in topography, result in micro-climatic variability, raise surface temperatures by up to 2 °C, may have more of an impact on malaria transmission than climate change alone [18–22] and therefore should be included in vulnerability and risk assessments.

### Vulnerability assessments in climate change and malaria research in East Africa

Vulnerability studies, which have long been affiliated with the disaster risk reduction and climate change adaptation communities [23, 24] are now increasingly used to map and interpret current and future risks related to climate change. Vulnerability is determined in part by human activities or interventions at the local level, which may, if successful, counteract the negative impacts of climate change. Furthermore, studies focused on projected increases in malaria transmission as a result of changes in climate should take into account the global decline of the disease by 60% from 2000 to 2015 mainly as a result of aggressive human interventions and treatment [25–27]. Therefore, a robust vulnerability assessment should not only take into account the impact of the climate-induced hazard to the population, but also the heterogeneity of the population and for malaria transmission, the differences in topography and hydrological characteristics of the landscape and other biological and socio-economic influences of transmission [16, 28–36] in a holistic and integrated manner [4, 6, 7]. Such an approach can incorporate an understanding of how changes in climate will impact the current burden of the disease (biophysical vulnerability). Moreover, this approach is also critical in identifying vulnerable populations and their capacity to respond (social vulnerability), taking into account other factors that affect the current burden of malaria and the effectiveness of current policies and programmes to manage the disease [37, 38].

Very few vulnerability assessments on climate change and malaria in East Africa are in published literature. A conceptual and methodological framework for modelling of social vulnerability for the East African region in a spatially explicit manner and independent of current disease prevalence, in order to provide options for targeted interventions was presented by Kienberger and Hagenlocher [39]. Risk and vulnerability was framed within the recent Inter Governmental Panel on Climate Change (IPCC) definition [40] in a dynamic and holistic manner and a number of related factors influencing disease risk were considered. Analysis and results established links to risk governance, climate change adaptation and relevant intervention strategies to several water-related vector borne diseases, including malaria. In a related study, Bizimana et al. [41] applied a composite indicator approach to assess social vulnerability to malaria transmission in Rwanda at a district level. An adapted vulnerability assessment framework [39] was used to identify indicators of different components of vulnerability in terms of generic susceptibility (i.e., lacking capacity to anticipate) and biological susceptibility (i.e., lacking capacity to cope or recover). Both studies mapped the main indicators of social vulnerability to malaria at district [41] and East African regional [39] levels. While both of these approaches provide useful tools for decision-making, only social vulnerability to malaria was considered. Also, there is an assumption of homogeneity of the population and landscape, which suggests uniformity of indicators while in reality there are differences in population and factors such as topography that will have an impact on the weight of indicators. Both papers acknowledge these limitations by suggesting that interventions should take into account the relevance of specific indicators of malaria vulnerability for different regions [39] and that future research should focus on an integrated vulnerability assessment that combines both environmental and social drivers [41].

Further research by Hagenlocher and Castro [42] addressed some of these limitations by modelling multi-dimensional vulnerability in Tanzania in a holistic and spatially explicit manner, using estimates of entomological inoculation rate (EIR) i.e. risk of infective bite as a proxy for malaria hazard. Causes of malaria risk and vulnerability were demonstrated to vary considerably across the country and risk, hazard and vulnerability maps that allow prioritisation of areas for malaria control were produced. By integrating malaria risk, vulnerability, and contributing factors in a holistic framework, evidence of issues that needed to be addressed locally to reduce malaria risk while accounting for variability within districts was provided. A useful output was an easily adaptable modelling framework however; limitations included incompatibility of the model with data that were not available in a spatially disaggregated format. This means that key vulnerability indicators such as acquired immunity to malaria, availability of malaria drugs, migration patterns, quality of the healthcare system, personal beliefs, behaviours and social networks were not included in the final model [42].

More recently, Bizimana et al. [43] integrated a set of weighted vulnerability indicators to define homogenous regions of social vulnerability to malaria in Rwanda. Although a useful approach in determining targeted interventions to specific high-risk areas and focus on factors that influence vulnerability, the model limitations did not allow for inclusion of key vulnerability indicators that did not have quantitative measurements such as social networks, migration and behavioural change. While these studies provide suitable frameworks for assessment of social vulnerability to malaria, none of them considered climate change/variability and the associated biophysical and social vulnerability at the same time. Biophysical and socio-economic factors are interdependent and must be considered simultaneously within an integrated systems framework, which assesses risk and vulnerability of communities to malaria. Therefore, using a systems approach, this paper builds on previous research to develop a framework for conducting an integrated risk and vulnerability assessment of the interplay among biophysical (especially climate change) and socio-economic and cultural factors on malaria transmission in East Africa.

## Methods

**Fig. 1:**
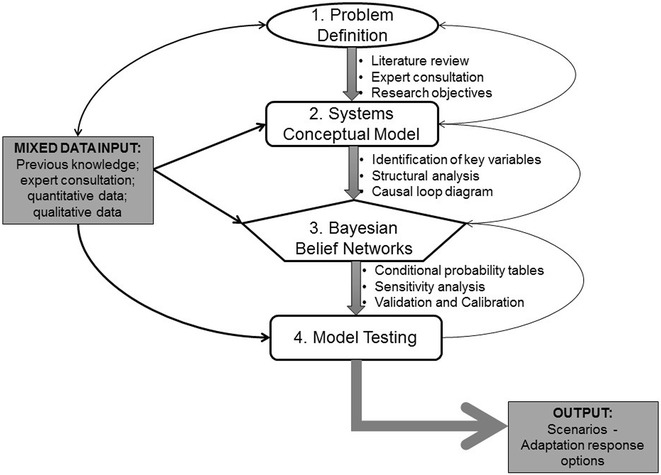
A flow chart showing the adapted systems modelling process

Building the systems model was an iterative process that involved problem definition and development of a conceptual model of the system under study. The model is then used to suggest the development and calibration of a Bayesian Belief Network (BBN) model. While there is not a standardized procedure for system’s modelling, there are some common steps as described by Sterman and Voinov [44, 45], which were adapted for our modelling process (Fig. 1). This approach captures contextual and expert knowledge of the system and then subjects the information to a structural analysis, which is used to formulate a conceptual model in the form of an influence diagram. This is the first step in developing a BBN model. BBN models are a useful method for undertaking scenario simulations because they can assimilate different kinds of data and information including qualitative social survey results, quantitative biophysical response functions, spatial environmental data, expert opinion and even missing data [43].

### Problem definition

This step involved an extensive literature review and expert consultations on the key variables and relationships involved in the climate change and malaria transmission cycle. Three key academics well versed in climate change, malaria transmission and climate change-malaria research in East Africa were contacted and consulted. The experts were provided with contextual information regarding the research and were interviewed on their knowledge of the connections between climate change and malaria transmission. Comprehensive reviews of climate change and malaria transmission have been covered in other papers [21, 46–48]. Some of these studies have developed suitable environmental, socio-demographic and behavioural indicators of malaria risk at regional, community and household levels [15, 21, 33, 48–50]. This previous knowledge and expert consultations were used to capture relevant knowledge about the system and to identify the relationships between key variables influencing risk of malaria infection in East Africa.

### Structural analysis

The malaria transmission cycle is a complex system with multiple non-linear and often interacting variables of climate change, environmental, biological and socio-economic influences thus, conceptualizing such a system is challenging. The cross-impact multiplication method (CIMM) [51] was used to undertake a structural analysis. The structural analysis method revealed key system components and interactions from a candidate set identified from the literature review and expert consultations and followed a four-step iterative process:

- Compilation of a candidate set of key variables from the literature review and expert consultations;
- Description of the relationships between variables based on contextual knowledge and expert opinion. The degree of influence between variables was rated 0 if there was no evidence of *direct influence* between two variables. Otherwise, the strength of the relationship was rated 1(low), 2 (medium), 3 (high) or 4 (potential);
- Identification of key variables using the CIMM approach which calculates the intensity of influence and dependency between variables; and
- The CIMM approach was also used to identify the relationships between the identified variables of the system through an analysis of the impact matrix by generating a map of direct influence, which separates the variables into four types according to degree of influence: (i) influential variables, which influence the system, but are not dependent on other variables; (ii) relay variables, which influence the system and are dependent on influential variables; (iii) dependent variables, which represent the system’s output variables and; (iv) autonomous variables, which are neither influential nor dependent and may or may not significantly affect the system depending on the strength of their relationships.

### Visualization of the systems conceptual model

After the structural analysis phase, an influence diagram (also known as a causal loop diagram or CLD) was constructed to visualize the key variables and interactions of the system). In an influence diagram, variables represent a stock of something or a quality of some kind that can increase or decrease. The variables are connected or linked by arrows that indicate a causal relationship; typically, a flow of information, energy or materials that cause a shift in the stock or quality of the affected variable. The direction of the arrow indicates the direction of causality while the polarity sign at the tip of the arrow (+ or −) indicates whether the relationship between the two variables is positive (increasing the effect) or negative (dampening the effect). The influence diagram was visualized using the software Vensim DSS for Windows Version 6.3.

### Developing the assessment framework

The systems conceptual model, represented by the influence diagram, complemented with existing risk and vulnerability assessment frameworks [21, 40] was used to develop an integrated assessment framework that considers both biophysical and social influences on malaria transmission. Multiple definitions exist for vulnerability, but for the development of our framework the definition of vulnerability in the context of vector-borne diseases as defined by [21] as “a combination of a change in exposure of humans to pathogens with environmental change and the sensitivity of the population to that change” was adopted. Also, the [21] definition of adaptive capacity as consisting of “…technologies, cultural tools and the public health infrastructure and resources that are available to implement appropriate management responses” was adopted. These definitions are consistent with those of IPCC Fifth Assessment Report, which defines vulnerability to disease and injury due to climate variability and climate change as “the propensity or predisposition to be adversely affected” dependent on generic (education, income, health status and responsiveness of government) and biological susceptibility (age, gender and immune status) [40, 52]. Finally, the risk of impacts from climate change was understood as “resulting from the overlap of hazards from the physical climate and the vulnerability and exposure of people, ecosystems, and assets” [40].

## Results

### Problem definition and identification of variables

The scope of this study was limited to the East African (Kenya, Tanzania, Uganda, Rwanda and Burundi) region based on researcher experience, existing networks and availability of previous extensive studies conducted in the region. The problem was therefore focused on determining how climate influences vulnerability to malaria transmission in East Africa. Based on the literature review and key insights from expert interviews, a candidate list of 36 variables were identified as important for understanding climatic impacts and malaria transmission cycle (Table 1) and grouped into two broad sets of biophysical and socio-economic indicators.

**Table 1:** Variables in climate change and malaria transmission identified from literature review and expert consultation

No	Variables	Description	Source
*Biophysical variables*			
1	Air temperature	Air temperature suitable for malaria transmission i.e. between 16 and 34 °C	[17, 32, 48, 57–61]
2	Water temperature	Mosquito habitat temperature suitable for breeding	[60, 62–64]
3	El-Nino	Periods of extreme rainfall	[14–16, 48, 65]
4	Average rainfall/precipitation	Mean monthly rainfall of at least 150 mm; rainfall season	[9, 17, 21, 31, 48, 61, 66, 67]
5	Relative humidity	Amount of water vapour present in air	[68–71]
6	Altitude	Height/distance above sea level	[36, 48, 69, 70, 72]
7	Micro-habitat changes	Changes in mosquito habitat micro-climate due to loss of forest cover or other environmental controls such as clearing of bushes	[63, 68–70, 73–78]
8	Topography	Physical land surface including hills and valleys, elevation	[33, 48, 79, 80]
9	Topographic wetness index	Percentage of ground water saturation of at least 5% for suitable mosquito breeding site	[30, 31]
10	Wetlands and water bodies	Proximity to swamps and other stagnant water bodies	[33, 63, 68, 74, 76, 77, 81]
11	Bare areas	Land without forest cover or other vegetation	[33, 82, 83]
12	Forest edge	Human proximity to forest boundaries and potential exposure to exposed mosquito breeding sites due to deforestation	[33, 62]
13	Agriculture	Land clearance, planting, livestock and maize farming, swamp drainage and farming, and water management i.e. water conservation using shallow wells, small-scale irrigation and creation of water drainage channels	[31, 33, 48, 49, 76, 77, 84–87]
14	Vector abundance	Increase in numbers of malaria mosquitoes	[32, 60, 82, 88]
15	Vector biting	Likelihood of an infective bite from a mosquito	[48, 70, 82]
16	Vector infection rate	Efficiency of transmission and infection with the malaria parasite by the mosquito	[48, 73, 82]
17	Vector adaptive behaviour	Changes in mosquito vector behaviour such as early biting or indoor resting	Expert input
18	Population under 5 years	Number of individuals under 5 years old	[48, 49, 74]
19	Immune status	Lowered immunity to malaria due to pregnancy or inexposure; acquired immunity to malaria from long term exposure	[48, 49, 79, 89, 90]
20	Interactions	Co-infections with other diseases such as HIV increase likelihood and severity of infection	[15, 50]
21	Drug resistance	Resistance of the malaria parasite to drugs/parasite evolution	[15, 48, 50]
*Socio-economic variables*			
22	Urbanisation	Expansion of urban areas and overcrowding in cities	[49]
23	Population migration/travel	Movement of people from low risk areas to malaria-endemic or epidemic-prone areas and vice versa	[48, 50]
24	Nutritional status	Poor health as a result of undernutrition or malnutrition	[48, 49]
25	Gender	Gender roles, expectations and cultural customs	[48, 49]
26	Poverty	Socio-economic conditions; household income, food and household assets	[15, 48, 49, 74]
27	Religious beliefs	Religion or superstitions in understanding or managing malaria and/or climate change impacts	[15, 49]
28	Perception	Knowledge and understanding of disease	[15, 33, 49]
29	Type of house	House with grass-thatched roof and mud walls (semi-permanent) or Bbrick house with tiled or aluminium roof (permanent); house with separate kitchen, house with ceiling and house with open eaves	[33, 48, 49]
30	Education level of household head	Education level of male or female head of household	[33]
31	Health-seeking behaviour	Willingness to seek treatment for malaria; households with malaria medicine in stock, self-medication, tradition/cultural norms and practices in malaria management	[48, 49]
32	Net use	Use of insecticide-treated bed nets to prevent malaria infection	[15, 33, 74]
33	Environmental controls	Keeping area around the houses cleared of shrubs and other overgrowth; safe disposal of plastics and other water-retaining containers	[15, 33]
34	Quality of health systems	Health services and policy; availability of health facilities; access to healthcare; quality of healthcare and capacity for malaria treatment	[15, 47, 48, 50]
35	Malaria vector control	Distribution and coverage of insecticide-treated bed nets by the government; coverage of households sprayed with malaria insecticide (indoor residual spraying)	[15, 48]
36	Quality of information	Reliable and easy to understand information systems for communicating weather and climate information or early warning systems for malaria epidemics	[15, 17, 50]

### Structural analysis

**Fig. 2:**
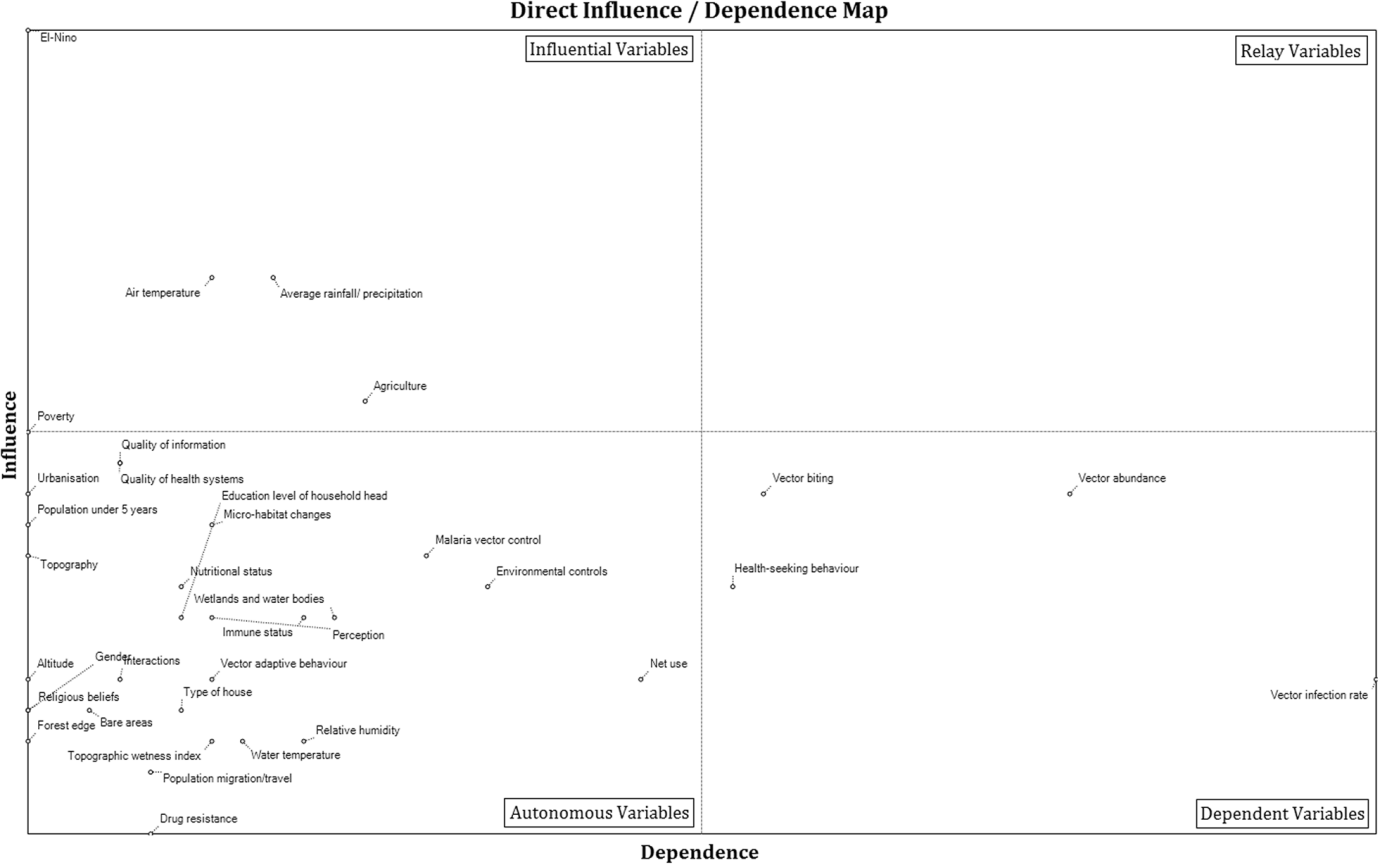
Direct influence-dependence map of variables of the climate change and malaria transmission system

The direct influence-dependence map generated from the structural analysis provided the key influential, relay variables, dependent variables and autonomous variables as shown in Fig. 2.

### Systems conceptual model of climate change and malaria transmission

The systems model was simplified by focusing mainly on the influential (El Nino, air temperature, average rainfall/precipitation, agriculture), dependent (vector biting, vector abundance, vector infection rate, health-seeking behaviour) and the autonomous variables with strong relationships within the system (water temperature, micro-habitat, topography, wetlands and water bodies, vector adaptive behaviour, population under five, immune status, poverty, education level of household head, nutritional status, perception, net use, malaria vector control, environmental controls, quality of information, quality of health systems).

**Fig. 3:**
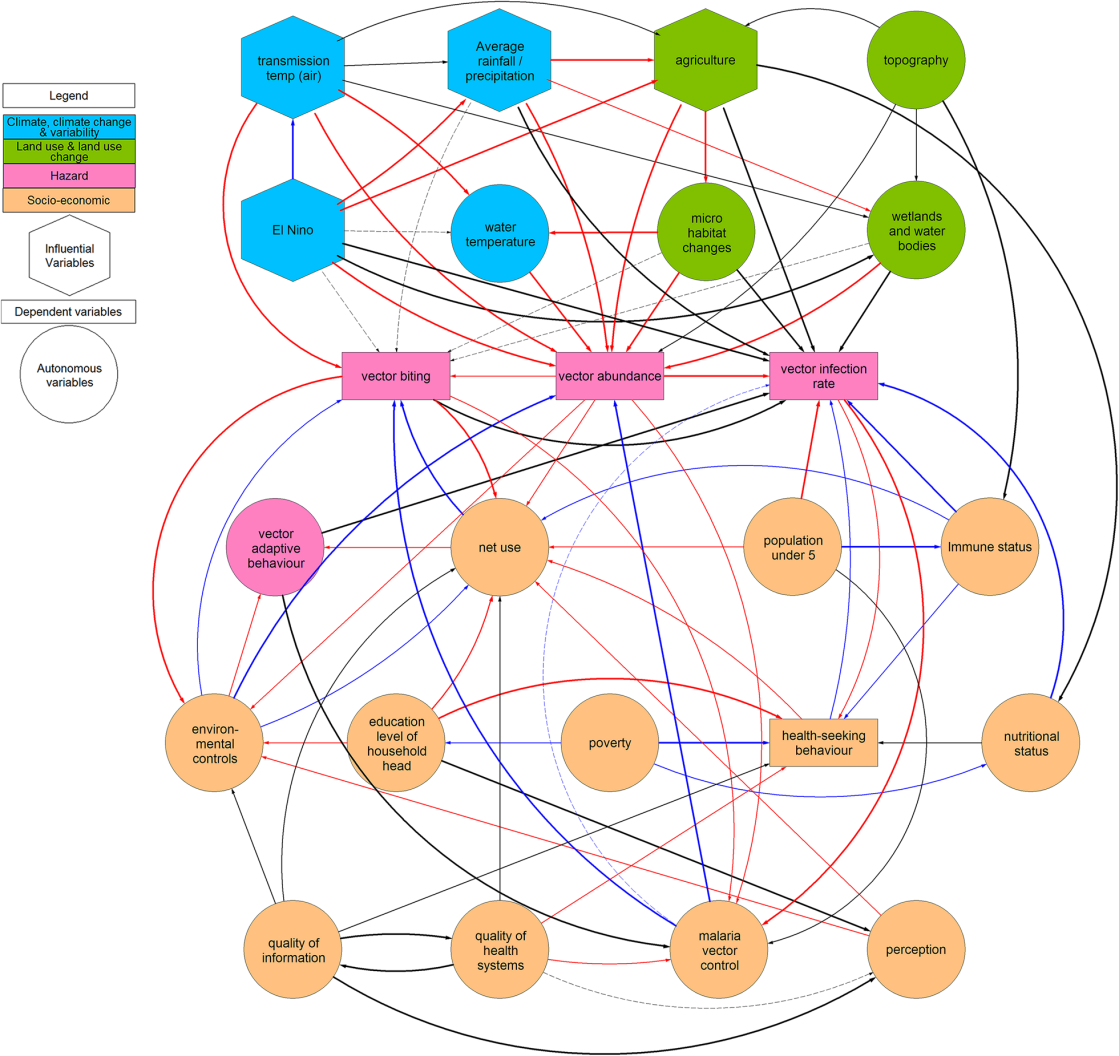
A systems conceptual model detailing the causal relationships between variables in the malaria transmission system

The visualization of the system conceptual model is illustrated in Fig. 3: Hexagons represent influential variables; rectangles are dependent variables; and circles are autonomous variables. The colours represent major classes of variables: blue = climate, climate change and variability variables; green = land use and land use change variables; and pink = malaria vector attributes; Additional to these biophysical variables are the socio-economic variables that are colour coded orange. The strength of relationships between variables is represented by solid lines (stronger relationships) and dashed lines (weaker relationships). The red arrows represent a positive relationship (+) (i.e., the recipient variable’s state or quality increases) between variables while blue arrows represent negative relationships (−). Lack of a polarity sign or black arrow indicates that the relationship can be either positive or negative or that the relationship has not been determined.

### An integrated risk assessment framework

**Fig. 4:**
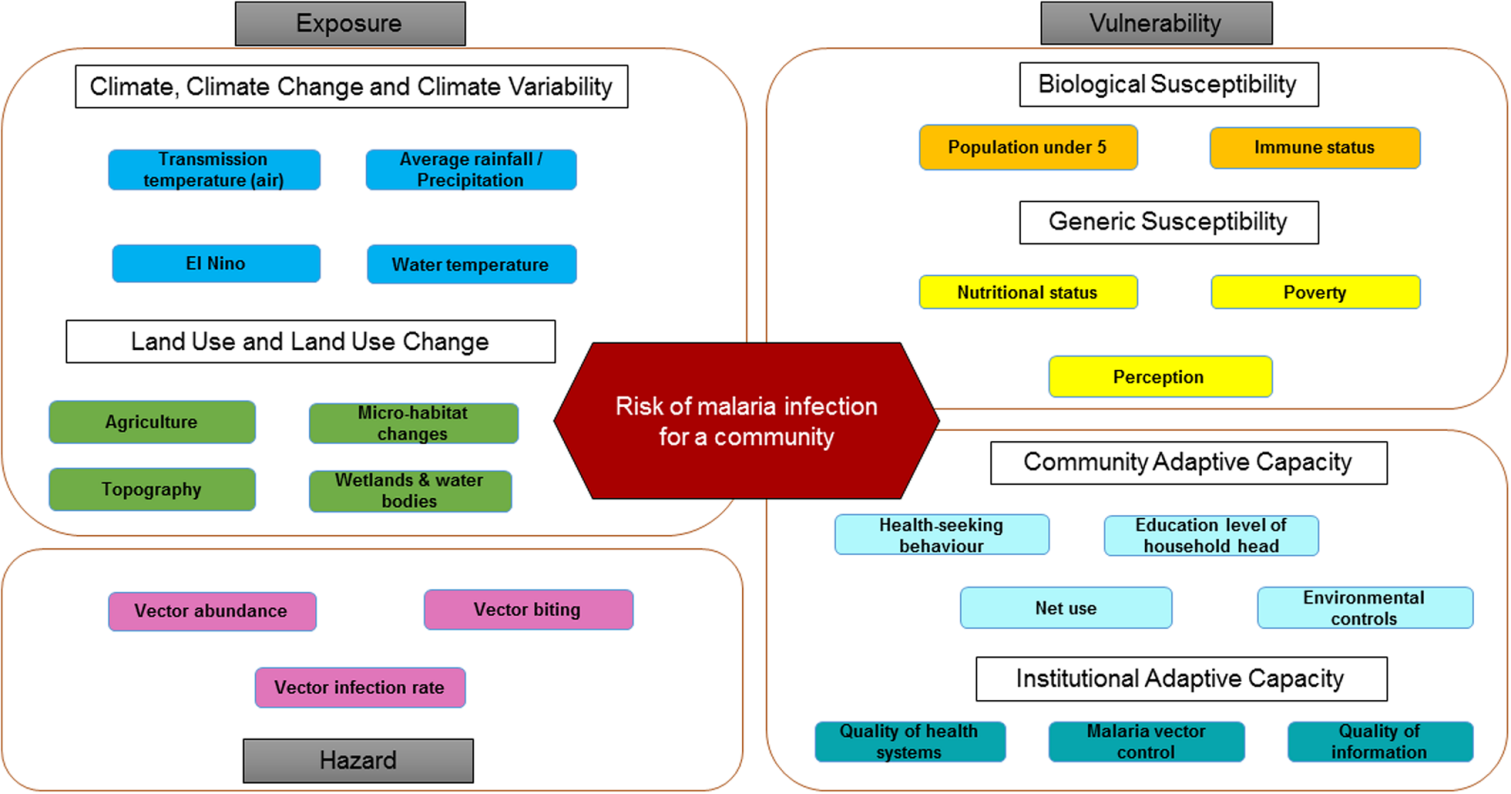
An integrated assessment framework to guide studies of climate change and malaria risk and vulnerability

The risk assessment framework (Fig. 4) was constructed based on the conceptual systems model. Risk of malaria infection was identified as the climate-related hazard, which is influenced by exposure to changes in climate, climate variability, land use and land use change and malaria vector attributes. Vulnerability to risk of malaria infection is determined by biological susceptibility, generic susceptibility and coping strategies at community and institutional levels. This assessment framework can be operationalised using BBN models as these allow for sensitivity analysis and exploration of the efficacy of policy recommendations under different scenarios. Sensitivity analysis can reveal the relative significance or leverage of driving variables, providing an objective basis to identify a subset of variables for model formulation.

## Discussion

Conducting integrated vulnerability assessments in climate change and malaria research is a difficult process due to the scarcity of empirical infection data on malaria, the multiple interacting determinants, the often indirect and non-linear causal chains, variations in exposure within affected populations and the high degree of model uncertainty [21, 37]. Previous studies have suggested integrated assessment frameworks of climate change and malaria. A framework that illustrated the environmental, socio-economic and biological factors affecting malaria incidence in the African highlands was proposed by Lindsay and Martens [47]. They defined this as an eco-epidemiologic modelling approach and reiterated the need for integrated modelling built on systems-oriented analyses that consider the interactions and feedback mechanisms between different sub-systems rather than treating them in isolation.

Another integrated assessment framework for infectious diseases (including malaria) was proposed by Chan et al. [46]. This framework presented a means by which cross-disciplinary research could be used to integrate the biologic, epidemiologic, ecologic and sociologic knowledge of climate impact on disease transmission in order to provide a reliable estimate of the climate-induced impact on the disease. Although this framework takes into account feedback loops resulting from human interventions and disease prevention, it did not consider which variables carry more weight in influencing transmission. Further research by Sutherst [21] presented a comprehensive review of global change and vector borne diseases, highlighting the complexity of malaria transmission and the major challenges involved in vulnerability assessments of the same. An integrated assessment framework was proposed for the study of these diseases and suggestions for future research included more studies that focused not just on vulnerability, but on designing adaptation options to changes in transmission risk using a systems approach.

Application of integrated vulnerability assessments have been rare, but are gaining prominence in literature; Dickin and Schuster-Wallace [53], modelled vulnerability to dengue in North-eastern Brazil using a water associated disease index (WADI) while Lyth and Holbrook [54], utilized systems thinking to undertake a quantitative and qualitative assessment of the complex social-ecological factors contributing to occurrences of ross river virus and impacting human health vulnerability in Tasmania. Studies in East Africa however, have been limited. A review of the literature identified only one study, which attempted to address both the biophysical and social influences of malaria transmission in East Africa within the context of climate change.

This study by Wandiga et al. [15], aimed to assess the vulnerability and coping capacity of target populations as well as the excess risk to which they are exposed as a result of climate change using both quantitative and qualitative methods. A positive relationship was demonstrated between changes in malaria cases and variations in monthly minimum and maximum temperatures. Other socio-economic factors such as drug misuse, inadequate knowledge of disease control, myths and superstitions at the household level that could contribute to higher incidences of malaria morbidity and mortality, were also identified. High poverty levels and weak health-care systems were determined as factors that reduce the coping capacity of the community. However, quantitative and qualitative data analyses were conducted separately and no sensitivity analysis was done thus, there is no determination of the relative contribution of any of these factors in increasing or decreasing the risk of malaria transmission.

More studies using integrated assessments of risk of climate-related diseases such as malaria are needed in East Africa. In this paper, some of the limitations of previous research have been addressed by presenting a systems-based conceptual model and assessment framework as the basis for a more integrated analysis of the risk of communities to malaria infection. In the suggested framework, this risk is influenced by exposure to changes in climate, climate variability, land use and land use change and malaria vector attributes. The integrated risk assessment framework can be used with established indicators of malaria transmission at household and community level [48–50].

The framework can be operationalized using BBN models, a task for future research. This will require using data from a mix of quantitative and qualitative sources including community and expert stakeholder surveys. Expert stakeholders will provide the necessary contextual knowledge while empirical knowledge from local stakeholders and the general community will be important in identifying specific determinants of vulnerability. Factors that increase exposure such as local geography and climate, social, economic and other environmental factors must also be taken into account. Sensitivity on the other hand, requires measures of abundance of the vector and pathogen on one hand and intrinsic immunity of the population on the other. Specific human behaviours that may reduce, increase or generate differential exposure should also be considered as part of adaptive capacity.

While BBN models have been widely applied in a range of natural resource management and decision support contexts [55, 56], there has been limited application of the same in climate change and public health assessments. Combining information from a range of quantitative and qualitative sources and integrating them using BBNs should result in a more robust model, informed by community needs and capabilities, leading to further knowledge generation and identification of targeted response strategies for decision-making and policy.

The framework presented is novel in the following aspects: (a) utilizing systems thinking to frame the problem of climate change and malaria transmission; (b) drawing upon mixed methods to integrate knowledge from different fields with stakeholder participation at different geographical scales and levels of governance; (c) its applicability in data-poor regions as narratives from stakeholders can complement quantitative data; and; (d) being sufficiently generic so that it can be applied to study impacts of climate change on other kinds of transmissible diseases.

A major limitation to the approach used primarily relates to the subjective way in which the candidate set of variables was identified. However, this process can be iterative with the results of the structural analysis being used to provide feedback to experts and stakeholders for further refinement. Additionally, BBN models have constraints including a limited ability to capture feedback loops from the response variable back to the drivers [55], therefore, the full suite of variables and interactions identified in our systems conceptual model and operational framework cannot be represented within a single BBN model. This can be overcome by constructing multiple BBN models that capture feedbacks and enable more detailed inspection of system subcomponents.

## Conclusions

It is important to remember that although climate change is the result of aggregate global emissions, climate change impacts vary regionally. Therefore, adaptation responses must always be tailored to local contexts and will be largely dependent on the risk and vulnerability profiles of communities. This vulnerability is context-specific and determined in part by human activities at the local level, which may far outweigh the influence of climate change. It follows that the same influencing factors for a developed country will not represent the same risks as those for rural communities in a developing country. Most vulnerability studies so far have been at global or regional levels and few have incorporated climate change, land use change and malaria transmission in an integrated manner at the community level.

The assessment framework presented here provides a first step approach for setting baselines against which risk can be assessed and for estimating the benefits of adaptation options for malaria risk management in East Africa. The approach is consistent with the current IPCC recommendations, which recognizes that risk of climate related hazards can occur due to many interacting influences and that focus should be on solutions for risk reduction. The framework can be applied at a community level using both quantitative and qualitative methods with stakeholder engagement and can be adapted to other data-poor regions with similar vulnerability profiles. The proposed use of BBN models with the framework would facilitate a robust assessment of contextually relevant and targeted adaptation strategies for dealing with malaria transmission, that incorporate both scientific and community perspectives.
